# Microbiological Quality and Presence of *Salmonella* spp. in Broiler Carcasses with and Without Visible Gastrointestinal Contamination During Industrial Processing

**DOI:** 10.3390/microorganisms13051124

**Published:** 2025-05-14

**Authors:** Bruno Leandro de Almeida Brito, Rafaela Assis Machado, João Luís Batista de Jesus, Francisco Yan Tavares Reis, Isabela Pádua Zanon, Monique Ribeiro Tiba Casas, Rodrigo Otávio Silveira Silva, Henrique César Pereira Figueiredo, Tadeu Chaves de Figueiredo, Marcelo Resende de Souza, Silvana de Vasconcelos Cançado

**Affiliations:** 1 Escola de Veterinária, Universidade Federal de Minas Gerais (UFMG), Avenida Antônio Carlos, 6627, Belo Horizonte 30123-970, Brazilrodrigo.otaviosilva@gmail.com (R.O.S.S.);; 2 Centro de Bacteriologia (NDEI), Instituto Adolfo Lutz, Avenida Doutor Arnaldo, 351, Pacaembu, São Paulo 01246-000, Brazil

**Keywords:** gastrointestinal contamination, chicken meat processing, pathogen

## Abstract

The industrial processing of broiler chickens has become increasingly automated to scale up meat production. However, certain procedures may cause rupture of the gastrointestinal tract, contaminating the products. The objective of this study was to evaluate the efficiency of the removal of visible contaminated broiler carcasses from the slaughter line based on their microbiological quality compared to non-contaminated ones. Carcasses were analyzed for *Escherichia coli* and aerobic mesophilic microorganisms counts, as well as *Salmonella* spp. detection. Carcasses with gastrointestinal contamination had significantly higher counts of aerobic mesophilic microorganisms and *E. coli* than those without contamination. However, carcasses without visible contamination also showed high counts of bacteria, indicative of the hygiene and sanitary concerns during slaughter. *Salmonella* spp. were detected in both types of carcasses, with no significant difference in the frequency of positive samples. The most frequently identified serovar was *Salmonella* Minnesota. The most frequently detected bacteria were *E. coli*, *Klebsiella pneumoniae*, *Citrobacter freundii*, and *Pseudomonas aeruginosa*. In conclusion, both contaminated and non-contaminated carcasses exhibited high bacterial counts, including potentially pathogenic microorganisms, highlighting the need for post-evisceration steps to reduce microbial contamination.

## 1. Introduction

Advancements in broiler slaughter technologies and procedures have been essential for improving the scale of poultry meat production. However, automated evisceration systems can lead to mechanical rupture of the gastrointestinal tract, resulting in contamination of carcasses, equipment, and surfaces by undesirable microorganisms [1,2].

The microbiota of the gastrointestinal tract in broiler chickens plays essential roles in digestion and immune system regulation. However, it can also harbor pathogenic microorganisms, such as *Salmonella* spp. and *Escherichia coli* [3].

The microbiological quality of poultry carcasses can be assessed by counting aerobic mesophilic microorganisms, which indicate the general hygienic conditions of processing. Additionally, testing for the presence of *E. coli* and *Salmonella* spp. serves as an indicator of sanitary conditions [3].

Outbreaks of gastroenteritis in humans have been linked to the consumption of chicken meat contaminated with *Salmonella*. In 2022, the European Union reported 65,208 cases of salmonellosis, leading to hospitalizations. *Salmonella* Enteritidis accounted for 67.3% of cases, followed by *S.* Typhimurium at 13.1% [4].

Therefore, the aim of this study was to evaluate the efficiency of the removal of visible contaminated broiler carcasses from the slaughter line based on the microbiological quality of them compared to non-contaminated ones by quantifying aerobic mesophilic bacteria and *E. coli*, as well as detecting *Salmonella* spp. and determining their serovars. Additionally, the isolated bacteria were identified using proteomic analyses.

## 2. Materials and Methods

### 2.1. Sampling

The carcasses were collected from a poultry slaughterhouse under official veterinary inspection. The facility uses an automated evisceration system capable of completely removing the viscera. Immediately after evisceration, 60 broiler carcasses were sampled, 30 with and 30 without visible gastrointestinal contamination on the external surface. Contamination from the entire gastrointestinal tract was considered, including the esophagus, crop, proventriculus, gizzard, and intestines. Sampling was conducted at the beginning, middle, and end of each shift. The collections took place over six weeks, with five carcasses from each category sampled per week. Each carcass was considered a separate replicate. Carcasses with bile contamination were excluded from the study.

### 2.2. Microbiological Analyses

After collection, each carcass was weighed and aseptically placed in a bag containing 1 mL of 0.1% buffered peptone saline solution per 10 g of carcass weight. The carcass was then rinsed for one minute with 35 inversion movements [5,6]. Aliquots of the rinsing liquid were serially diluted from 10^−1^ to 10^−6^ for the enumeration of aerobic mesophilic microorganisms and *E. coli*, with results expressed in colony-forming units (CFU) per gram.

Aerobic mesophilic microorganism counts were performed following the ISO 4833-1 methodology [7]. The plates were incubated at 32 ± 2 °C for 72 h. *E. coli* enumeration was conducted by inoculating sample dilutions onto Petrifilm^®^ plates (3M™, St. Paul, MN, USA) [8], which were incubated at 35 ± 2 °C for 48 h.

Detection of *Salmonella* spp. was carried out according to ISO 6579-1 [9]. Aliquots of 25 mL of rinse liquid from each carcass were transferred to 225 mL of 1% peptone saline solution and incubated at 36 ± 2 °C for 18 h. Subsequently, 1 mL was transferred to 10 mL of Muller-Kauffmann Tetrathionate-Novobiocin (MKTTn) broth (Oxoid, Basingstoke, Hampshire, England, UK) and incubated at 36 ± 2 °C for 24 h. Additionally, 0.1 mL was transferred to 10 mL of Rappaport-Vassiliadis broth with soy (RVS) (Hexis, Jundiaí, São Paulo, Brazil) and incubated at 41.5 ± 2 °C for 24 h.

After incubation, aliquots from both MKTTn and RVS broths were streaked onto xylose lysine deoxycholate (XLD) agar (Titan Biotech, New Delhi, India) and Brilliant Green Phenol-Red Lactose Sucrose (BPLS) agar (Oxoid). The plates were incubated at 36 ± 2 °C for 24 h.

### 2.3. Identification of Salmonella Serotypes

Initially, all isolates were confirmed as *Salmonella* spp. using conventional biochemical tests. Subspecies determination was based on additional biochemical characteristics [10]. *Salmonella* serotyping was conducted according to the 9th edition of the White–Kauffmann–Le Minor scheme [11], using agglutination tests with antisera targeting somatic (O) and flagellar (H) antigens. The antisera were prepared at the Laboratory of Enteric Pathogens, Adolfo Lutz Institute (São Paulo, Brazil).

### 2.4. Proteomic Identification of Microorganisms

For identification of other microorganisms, colonies grown on PCA, BPLS, and XLD with distinct morphological characteristics were identified using Matrix-Assisted Laser Desorption Ionization—Time of Flight (MALDI-TOF) mass spectrometry with Microflex^®^ equipment (Bruker Daltonics, Bremen, Germany). Each selected colony was transferred to a stainless-steel target plate, followed by the addition of 1 µL of 70% formic acid and 1 µL of α-cyano-4-hydroxycinnamic acid before being processed. Prior to measurement, calibration was performed using a bacterial test standard (*Escherichia coli* DH5 alpha; Bruker Daltonics).

The mass spectrum obtained at each analysis, based on the bacterial ribosomal protein profile, was compared with the database of the manufacturer and analyzed using the MALDI Biotyper Compass Version 4.1.100, with the library BDAL V13.0.0.2 (Bruker Daltonics). Identification scores were interpreted according to the manufacturer’s criteria: scores ≥ 2000 indicated species-level identification, scores between 1700 and 2000 indicated genus-level identification, and scores below 1700 were considered unreliable for identification [12].

### 2.5. Experimental Design

The experiment followed a randomized block design with two treatments (contaminated and non-contaminated carcasses) and six blocks (weeks), with 30 replicates, each consisting of one carcass. Homoscedasticity was assessed using the Levene test. The counts of aerobic mesophilic microorganisms and *E. coli* were found to be nonparametric and were transformed into log₁₀ values. Statistical analyses were conducted using ANOVA, with the Fisher test applied at a 5% significance level. The frequency of *Salmonella* spp. positivity was compared using the Chi-square test. SAS (Statistical Analysis System version 9.4) software was used for data management and statistical analysis.

## 3. Results and Discussion

### 3.1. Counts of Microorganisms Indicating Sanitary and Hygienic Quality

Carcasses with gastrointestinal contamination had significantly higher (*p* < 0.05) counts of aerobic mesophilic microorganisms and *E. coli* compared to those without contamination (Table 1). Elevated counts of aerobic mesophilic microorganisms indicate hygienic deficiencies during the slaughter process, while higher *E. coli* levels suggest sanitary inadequacies, as this bacterium is part of the enteric microbiota of birds.

There is limited information in the literature regarding the influence of gastrointestinal contamination on the microbiological quality of chicken carcasses collected immediately after evisceration. In this study, both contaminated and non-contaminated carcasses exhibited high counts of aerobic mesophilic microorganisms and *E. coli*, suggesting the occurrence of cross-contamination. Cibin et al. [13] reported high microbial counts in broiler carcasses contaminated with gastrointestinal content, showing that those parameters indicate the sanitary conditions of the processing.

Quality Control Programs establish the absence of visible gastrointestinal contamination as a critical limit for hygienic and sanitary safety. However, during slaughter, carcasses with high microbial loads but no visible gastrointestinal content are not excluded from processing.

The contamination of chicken carcasses presents a challenge for inspection services, as carcasses with high microbial loads but no visible contamination may go undetected. As a result, foods derived from these carcasses may enter the market, posing a risk to consumer health.

### 3.2. Salmonella spp. Detection

*Salmonella* spp. was detected in 23 (38.4%) of the 60 analyzed carcasses. The pathogen was found in 10 (33.3%) of the 30 carcasses with visible gastrointestinal contamination and in 13 (43.3%) of those without. Chi-square analysis showed no significant difference (*p* > 0.05) in *Salmonella* positivity between the two groups. Jimenez et al. [14] also did not observe differences in the frequency of these bacteria when comparing carcasses with and without gastrointestinal contamination.

Out of 612 colonies with suggestive morphotypes from BPLS and XLD media, 60 were confirmed as *Salmonella* spp., with 22 (44%) originating from carcasses with visible contamination and 28 (56%) from those without. Cross-contamination between carcasses, surfaces, equipment, and handlers may contribute to the spread of *Salmonella* spp. in the poultry slaughter environment.

The similarity in sample positivity between treatments suggests that visible gastrointestinal contamination is an inadequate parameter for identifying carcasses positive for *Salmonella* spp.

### 3.3. Identification of Salmonella Serotypes

Of the 48 colonies positive for *Salmonella* spp., the following serotypes were identified: Minnesota (n = 25, 52%), Anatum (n = 8, 17%), Ealing (n = 5, 11%), Mbandaka (n = 4, 8%), Agona (n = 2, 4%), Corvallis (n = 1, 2%), Rissen (n = 1, 2%), Soerenga (n = 1, 2%), and Schwarzengrund (n = 1, 2%). Studies that assessed the presence of *Salmonella* spp. in chicken carcasses also identified the Minnesota serotype at frequencies of 31.4% [15] and 25% [16]; Anatum of 66.7% [17] and 13.7% [18]; and Ealing of 0.4% [19].

Although Brazilian health authorities are particularly concerned about the Typhimurium and Enteritidis serotypes, the detection of *Salmonella* Minnesota is also alarming. As demonstrated by Habib et al. [16], this serotype, isolated from chicken meat sold in the United Arab Emirates, exhibited multidrug resistance to several antimicrobials, including colistin.

Lin et al. [20] and Boubendir et al. [21] demonstrated that the evisceration stage poses a significant risk for carcass contamination by *Salmonella* spp. and that contaminated carcasses serve as a primary source of cross-contamination.

*Salmonella* spp. are recognized as one of the leading causes of foodborne illnesses worldwide [22]. Epidemiological studies identify chicken meat as the primary source of *Salmonella* spp. transmission to humans [23], given that birds serve as a significant reservoir for these bacteria [24]. Although the serotypes identified in the present study are not *Salmonella* Enteritidis or *S.* Typhimurium, non-typhoidal serotypes still possess invasive capabilities and can cause systemic infections [25], leading to substantial public health and economic challenges [26].

### 3.4. Identification of Contaminating Microorganisms

**Figure 1:**
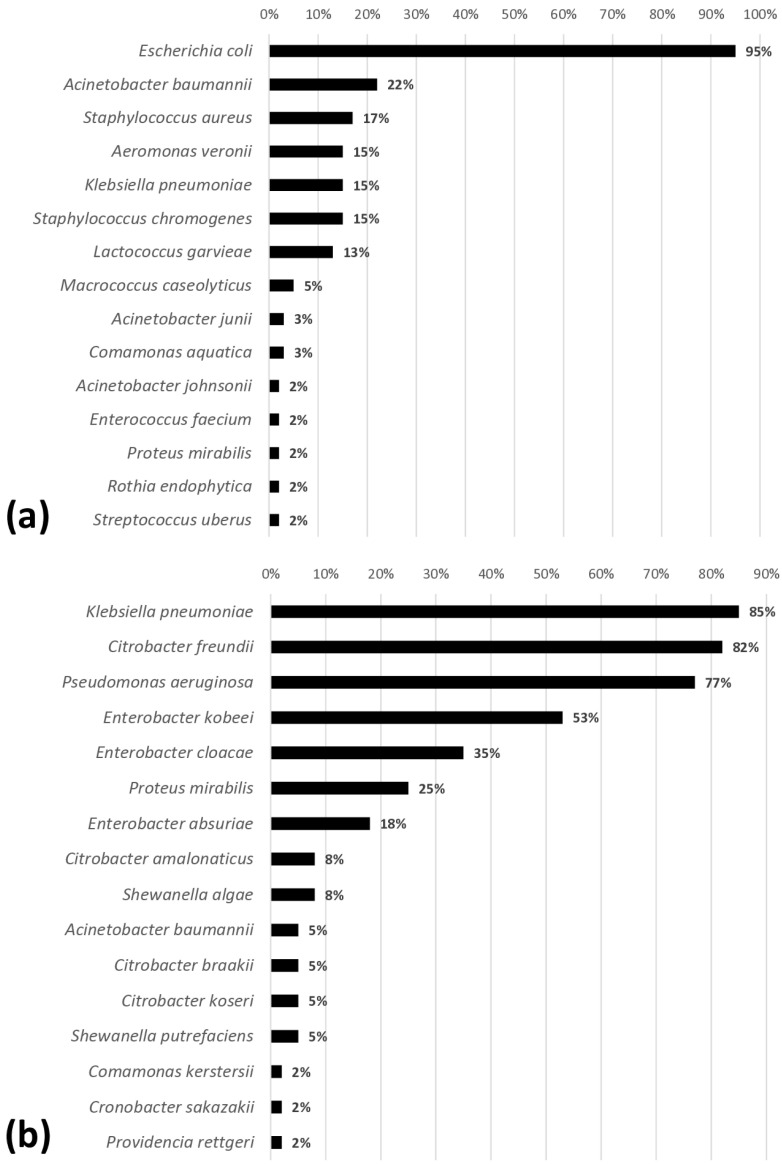
Frequencies of microorganisms isolated in broiler carcasses (n = 60) and identified by MALDI-TOF. (**a**) Isolated from PCA agar; (**b**) isolated from XLD or BPLS agar.

Of the 1220 colonies with distinct morphotypes, 30 genera, including 49 species of bacteria, were identified (Figure 1). The carcasses were predominantly contaminated with Gram-negative bacteria, including *E. coli* (95%), *Klebsiella pneumoniae* (85%), *Citrobacter freundii* (82%), *Pseudomonas aeruginosa* (77%), and *Enterobacter kobei* (53%).

The microbiota of broiler carcasses, including both spoilage and pathogenic bacteria, depends on the occurrence and extent of contamination, as well as the exchange of microorganisms between carcasses [27]. In this study, the high frequency of *E. coli* can be attributed to its presence as part of the intestinal microbiota of birds, in addition to the potential for slaughter stages to cause rupture of the viscera and contaminate the carcasses. Results of carcasses contaminated with *E. coli* were also reported by Hussain et al. [28] with 78%, Davis et al. [29] with 87.6%, Gautam et al. [30] with 100%, and Adzitey et al. [31] with 80%. This also explains the high frequency of other bacteria from the *Enterobacteriaceae* family, such as *Citrobacter* and *Enterobacter*, found on the sampled carcasses.

*Klebsiella pneumoniae* poses a potential risk to humans due to the severity of the associated conditions, which include urinary tract infections, meningitis, pneumonia, and septicemia [32,33]. The presence of this pathogen was reported by Mourão et al. [34] at various points along the poultry production chain, including one-day-old chicks (7%), cleaned poultry houses (36%), pre-slaughter broiler fecal samples (79%), and broiler carcasses (50%).

*Pseudomonas aeruginosa*, although not a member of the Enterobacteriaceae family, is part of the gastrointestinal microbiota of poultry and has also been reported as an opportunistic pathogen in these animals, being associated with cases of septicemia and diarrhea, as well as causing respiratory symptoms [35].

## 4. Conclusions

The presence of gastrointestinal contents in broiler carcasses is not a sufficient indicator of safety during processing, as carcasses without visible contamination may still harbor high bacterial counts, including potentially pathogenic microorganisms. Proper sanitary procedures are essential to reduce contamination during the slaughter stages and ensure the production of safe food.

## Figures and Tables

**Table 1:** Means and standard deviations of aerobic mesophilic microorganism and *E. coli* counts (Log_10_ CFU/g) in 60 broiler carcasses, with and without visible gastrointestinal contamination, collected immediately after automated evisceration.

	Counts of Bacterial Indicators	
Type of Carcass	Aerobic Mesophilic Microorganisms	E. coli
Without visible gastrointestinal contamination	5.42 ± 0.40 ^b^	3.74 ± 0.68 ^b^
With visible gastrointestinal contamination	6.01 ± 0.61 ^a^	4.70 ± 0.62 ^a^

Means followed by distinct letters in the same column differ by the Fisher test (*p* < 0.05).

## Data Availability

The raw data supporting the conclusions of this article will be made available by the authors on request.
